# Ischemic Portal Venous Gas and Pneumatosis Intestinalis Managed Conservatively in a Patient with Rheumatic Heart Disease and Warfarin Induced Coagulopathy: A Case Report

**DOI:** 10.31729/jnma.8302

**Published:** 2023-10-31

**Authors:** Prajwal Dahal, Sharma Paudel, Rakesh Kumar Sah, Sabina Parajuli, Kiran Kayastha

**Affiliations:** 1 Department of Radiology and Imaging, Grande International Hospital, Tokha, Kathmandu, Nepal; 2 Department of Radiology and Imaging, Tribhuvan University Teaching Hospital, Maharajgunj, Kathmandu, Nepal; 3 Department of General Surgery, Grande International Hospital, Tokha, Kathmandu, Nepal; 4 Department of Pathology, National Academy of Medical Sciences, Bir Hospital, Mahaboudha, Kathmandu, Nepal; 5 Grande International Hospital, Tokha, Kathmandu, Nepal

**Keywords:** *case reports*, *mesenteric ischemia*, *portal vein*, *rheumatic heart disease*, *warfarin*

## Abstract

Mesenteric ischemia is a surgical emergency. The presence of hepatic portal venous gas and pneumatosis intestinalis is a frequent finding in computed tomography. Not all hepatic portal venous gas and pneumatosis intestinalis are due to mesenteric ischemia. A 70-year-old female, a known case of diabetes mellitus, rheumatic heart disease and atrial fibrillation under warfarin presented with diffuse abdominal pain, multiple episodes of vomiting and ecchymosis in bilateral flanks. Evaluation of the coagulation profile suggested warfarin-induced coagulopathy. Portal venous gas was detected during an ultrasound examination. Subsequent contrast-enhanced computed tomography abdomen showed hepatic portal venous gas, pneumatosis intestinalis, paucity of branches of the ileocolic artery, and reduced enhancement of caecum and ascending colon. Mild ascites was present in the pelvis. Arterial blood gas analysis revealed compensated metabolic acidosis. The patient was managed conservatively and discharged after nine days of hospital admission. Conservative approach can be considered for suspected mesenteric ischemia in surgically unfit candidates.

## INTRODUCTION

Portal venous gas (PVG) is characterized by air within branches of the intrahepatic portal vein or main portal vein and its tributaries.^1^ Pneumatosis intestinalis (PI) is characterized by air in the intramural and submucosal plane of the gut wall.^1^ The causes of PI and PVG might be benign to life-threatening.^2^ Benign causes include intestinal obstruction, sepsis, inflammatory bowel disease, collagen vascular disease, etc.^3^ PVG and PI in the setting of ischemic pathology with or without visible thrombus are considered fatal if emergency surgery is not performed.^2^ Here, we present a case of ischemic PI and PVG managed conservatively in a patient with warfarin-induced coagulopathy.

## CASE REPORT

**Figure 1:**
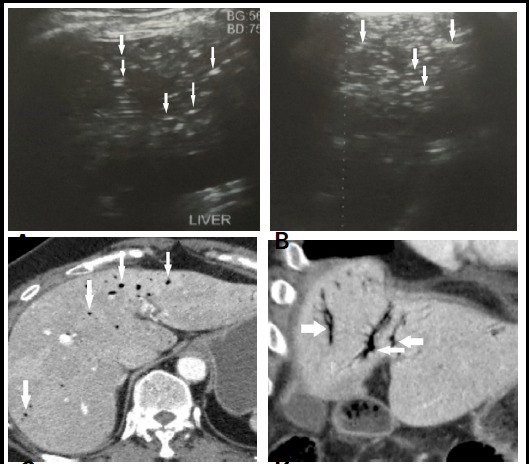
A), B) Ultrasound images showing air in intrahepatic portal vein branches. C) CECT images showing air in the intrahepatic portal vein branches.

A 70 years old female, a known case of diabetes mellitus, rheumatic heart disease (severe mitral stenosis, mild mitral regurgitation and severe tricuspid regurgitation) with atrial fibrillation and pulmonary arterial hypertension under 4 mg warfarin daily dose presented with diffuse abdominal pain, multiple episodes of vomiting and ecchymosis in bilateral flanks. Vitals were stable. On examination, the abdomen was tender and the bowel sound was absent. Prothrombin time was 120 s and the international normalized ratio (INR) was 9.23 suggesting warfarin-induced coagulopathy. White blood count was towards the higher side (11070 cells/mm^3^). Compensated metabolic acidosis was present (pH was 7.4, bicarbonate level was 18.8 mmol/L and partial pressure of carbon dioxide level was 27.7 mmHg). Serum lactate was normal in arterial blood gas (ABG) analysis. Hepatic portal venous gas (HPVG) was detected during an ultrasound examination and subsequent contrast-enhanced computed tomography (CECT) abdomen showed gas in intrahepatic branches of the portal vein (Figure 1).

**Figure 2:**
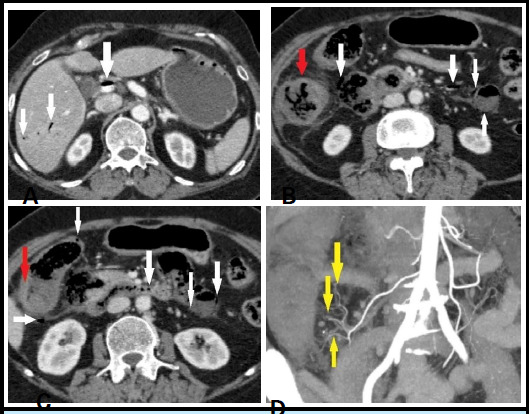
A) CECT abdomen showing air in main portal vein. B), C) Intramural air in duodenum, proximal jejunum, caecum, ascending colon, tributaries of the portal vein with diffuse thickening and reduced enhancement of ascending colon, D) Paucity of branches of ileocolic artery.

On the CECT abdomen, presence of gas in the main portal vein, superior mesenteric vein and tributaries of portal vein draining the duodenum, proximal jejunum, caecum and ascending colon with diffuse thickening and reduced enhancement of the caecum and ascending colon were seen. There was a paucity of branches of the ileocolic artery. Mild soft tissue stranding was seen around the caecum and ascending colon. Mild fluid was seen in the pelvis. Diagnosis of mesenteric ischemia was made (Figure 2).

**Figure 3:**
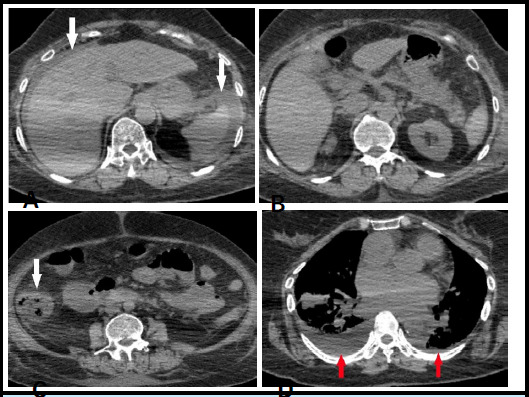
A), B) Resolution of PVG and PI and mild ascites, C) Thickening of the caecum, and ascending colon is still present, D) Mild bilateral pleural effusion is present.

Due to the presence of valvular heart disease and a deranged coagulation profile, the patient was declared unfit for surgery by the anaesthesia team. Since the patient was hemodynamically stable, serum lactate level was within normal range and the patient was declared unfit for surgery, a decision of conservative management was made. The patient was kept nil per oral and given broad-spectrum antibiotics (piperacillin, tazobactam, metronidazole) and warfarin stopped for a few days. The patient complained of melena the next day. However, the abdominal pain was subsided. There was a progressive clinical improvement of the patient. There was resolution of HPVG and PI in plain computed tomography (CT) done after 4 days Diffuse thickening of the caecum and ascending colon was still present. Ascites had increased and mild bilateral pleural effusion was seen (Figure 3).

Since the patient was hemodynamically stable and clinically improving, conservative management was continued. Warfarin was started again after 7 days. The patient was discharged after 9 days of admission and on follow-up, the patient is doing well 3 months after the incident.

## DISCUSSION

HPVG was described in neonates with necrotising enterocolitis.^2^ First differential diagnosis of HPVG is pneumobilia.^4^ The differentiating feature is the presence of air in the periphery of the liver (within 2 cm of the liver capsule) in HPVG and in the central part of the liver in pneumobilia due to the direction of flow in the portal vein and bile duct.^4^ The reported incidence of PI is 0.03%.^2^ With the increasing use of CT for abdominal imaging, the detection of PI is increasing these days.^2,3,5^ Most patients with PI in abdominal CT are between 30 to 50 years old and are usually asymptomatic.^2^ Pathophysiology of PI and HPVG is unclear; however, two widely accepted theories are mechanical theory and bacterial translocation.^3^ According to mechanical theory, PI is seen due to gas migration from the intestinal lumen. Bacterial translocation is defined as the presence of bacteria or its toxin in the wall of the intestine or tributaries of the portal vein.^1^ The migration of gas and translocation of bacteria occur due to a breach in the mucosal barrier which occurs in conditions like mesenteric ischemia, bowel obstruction, inflammatory bowel disease, sepsis, abdominal abscess, diverticulitis, malnutrition, drugs like alpha-glucosidase inhibitor etc. Mortality of ischemic HPVG and PI is considered 100% if surgical intervention is not done. However, patients who survived mesenteric ischemia with conservative management. has been described.^1,5^ An algorithm for risk assessment and management of HPVG and PI has been developed.^5^ Scores are given for different clinical and laboratory findings. A total score less than 4 is suggestive of benign cause. A total score of 6 and above is suggestive of mesenteric ischemia. The total score between 4 and 6 suggests the possibility of mesenteric ischemia.

In our case, the picture seen in CT in the background of rheumatic heart disease and warfarin-induced coagulopathy is highly suggestive of mesenteric ischemia. The ischemia is likely due to an embolic shower of small thrombi. We propose our patient had reversible mesenteric ischemia rather than bowel necrosis. There was spontaneous dissolution of microthrombi and circulation was restored and conservative management became successful. According to an algorithm for risk assessment and management,^5^ our patient's score is 6.5 which is also highly suggestive of mesenteric ischemia. We had no other alternative than conservative management. Some might argue that a normal lactate level indicates the absence of necrosis. However, a few cases are published where they describe conservative management of acute mesenteric ischemia and in their cases, lactate level is within normal limits.^1,5^ Literature has described conservative management of mesenteric ischemia with hyperbaric oxygen, urokinase, antibiotics, and total parental nutrition yield good results.^2^ However, we managed the patient with antibiotics and gave rest to the bowel.

Not all HPVG and PI are mesenteric ischemia. The decision to operate should be taken on the basis of the clinical condition of the patient rather than imaging alone. In a small number of patients who are unlikely to survive laparotomy, conservative management can be attempted for suspected acute mesenteric ischemia.
